# Supplementary figures and images for: Prediction of cancer cell line-specific synergistic drug combinations based on multi-omics data
Source: PeerJ. 2025 Feb 25;13:e19078. doi: 10.7717/peerj.19078 (PMC11869890; doi:10.7717/peerj.19078)

A

Drug pairs-Cell line Distribution

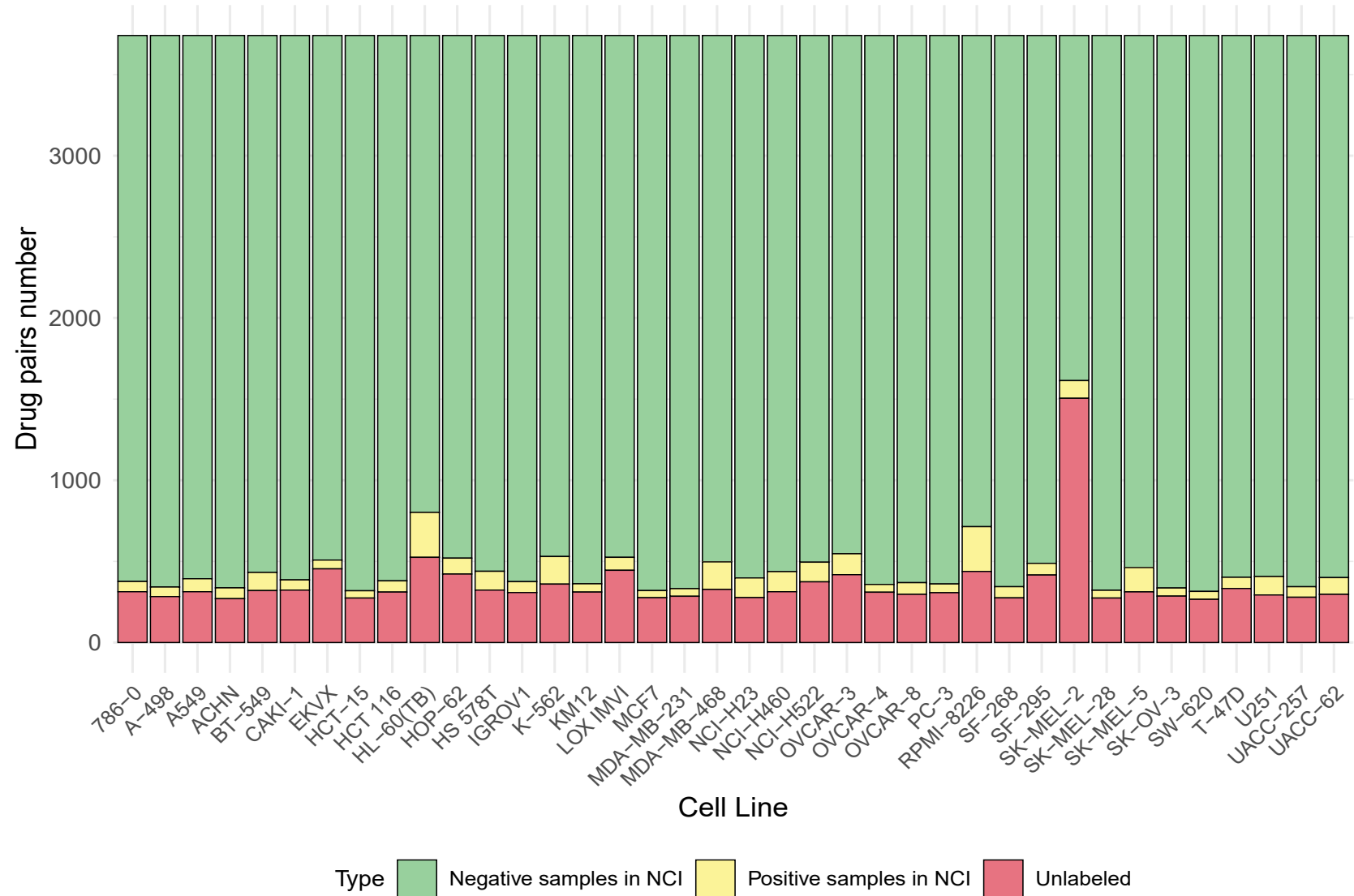

B

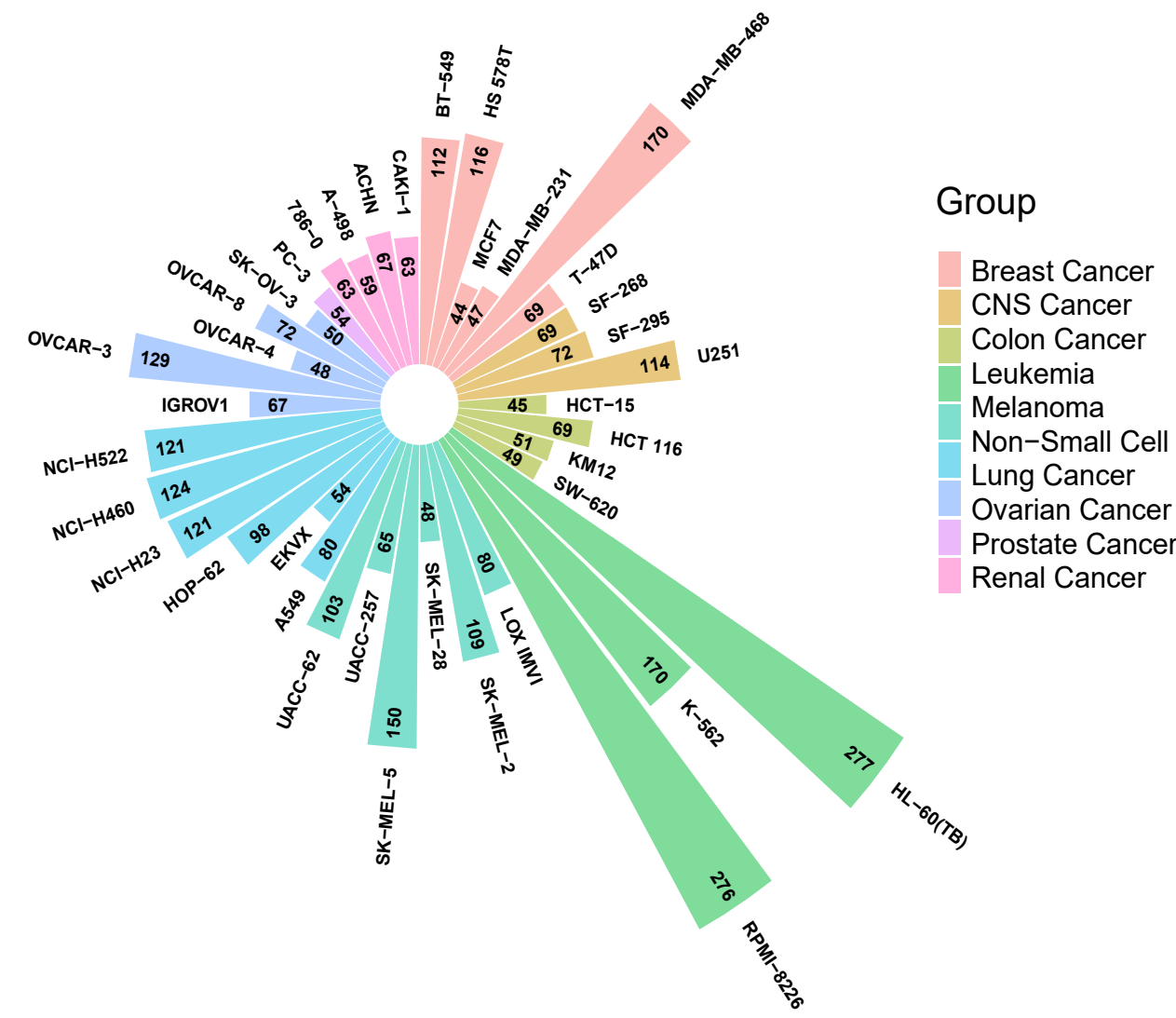

Supplement: Supplemental Information 1 — (A) The stacked bar chart illustrates the number of drug pairs in different cell lines, taking the threshold of 10 as an example. Green represents the number of drug pairs without synergistic effects according to NCI, yellow represents the number of drug pairs with synergistic effects according to NCI, and red represents the number of untested drug pairs in the validation set by NCI. (B) Positive drug pair counts in cell lines corresponding to the 9 types of cancer when the threshold is set to 10. [file peerj-13-19078-s001.pdf]

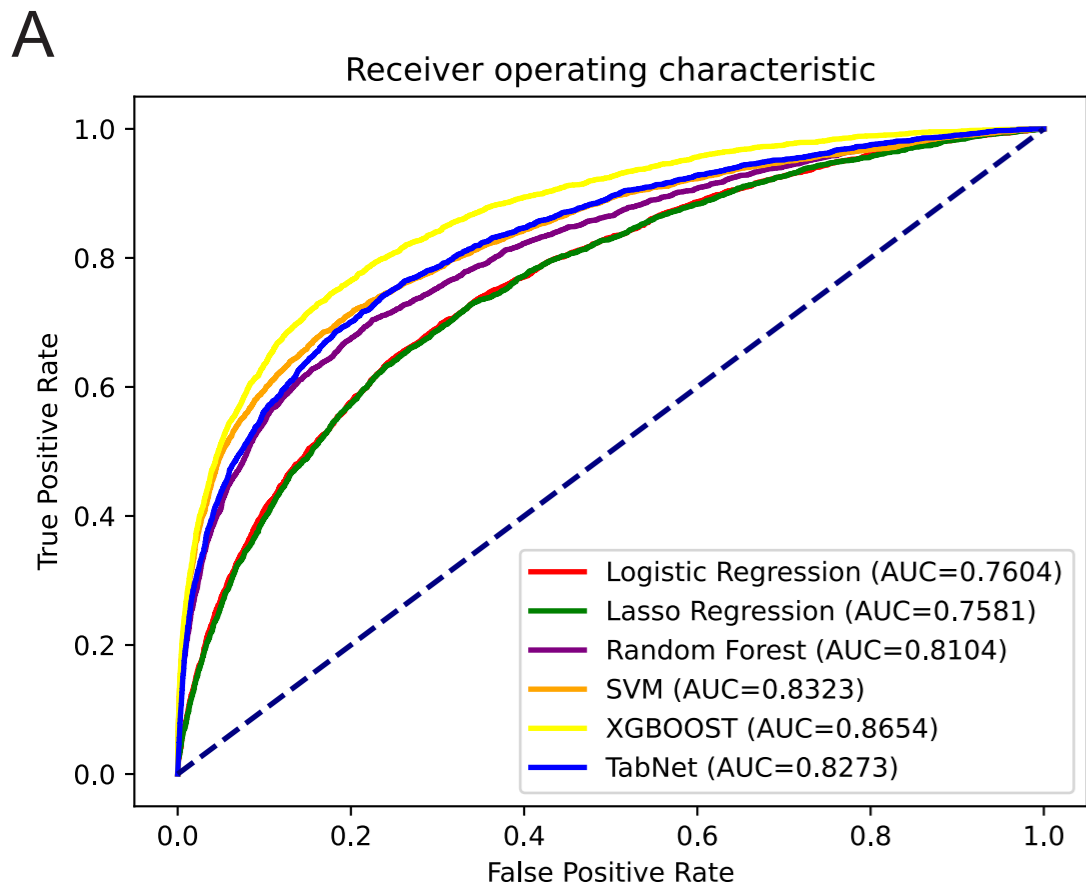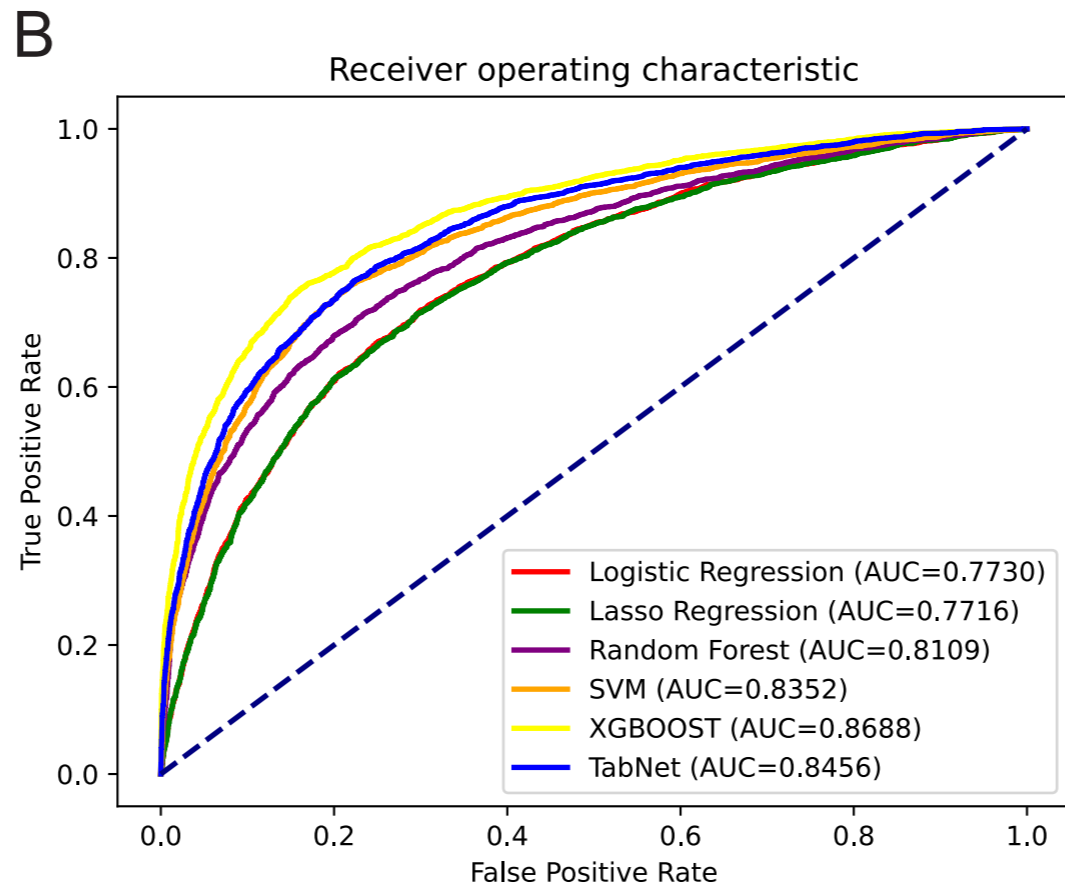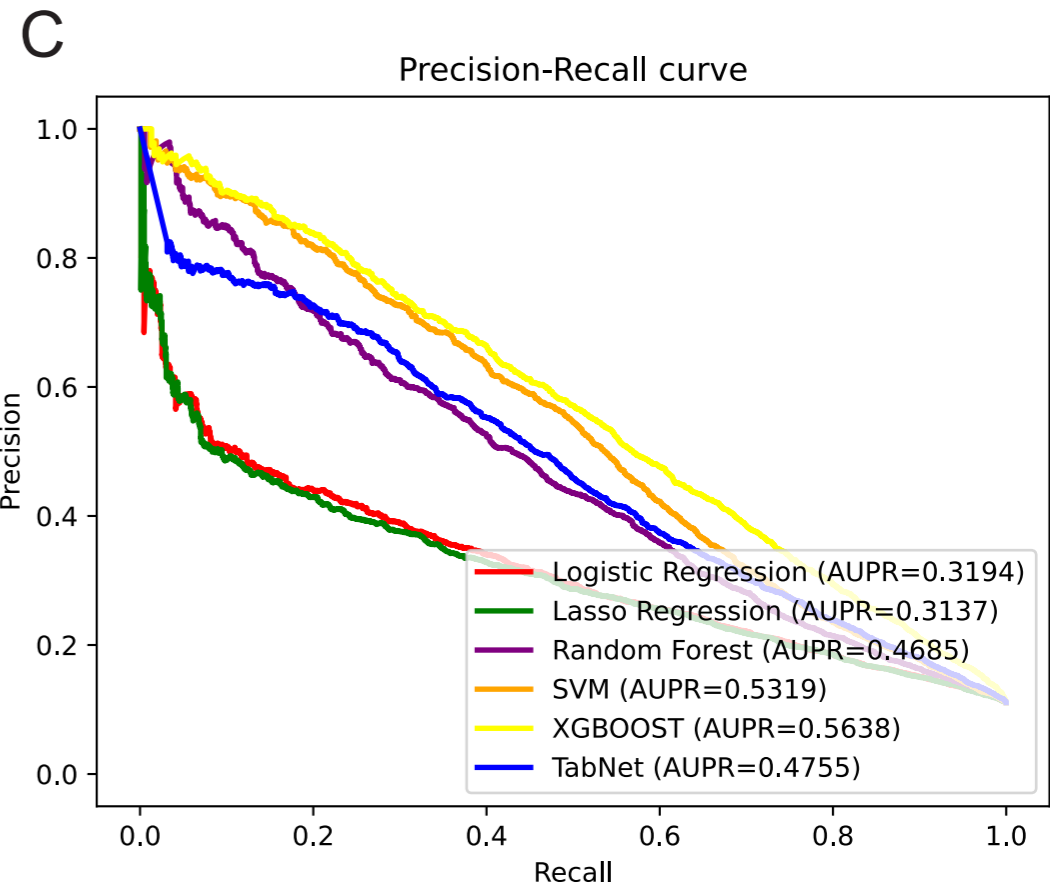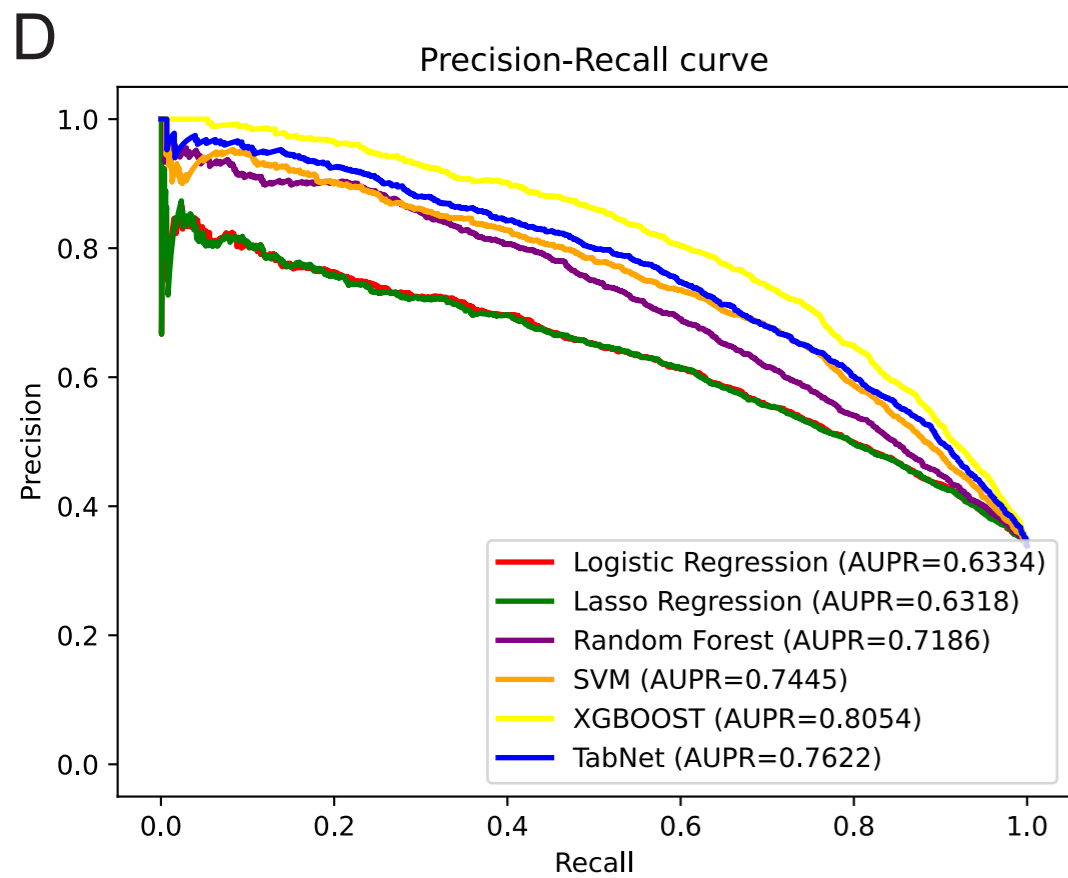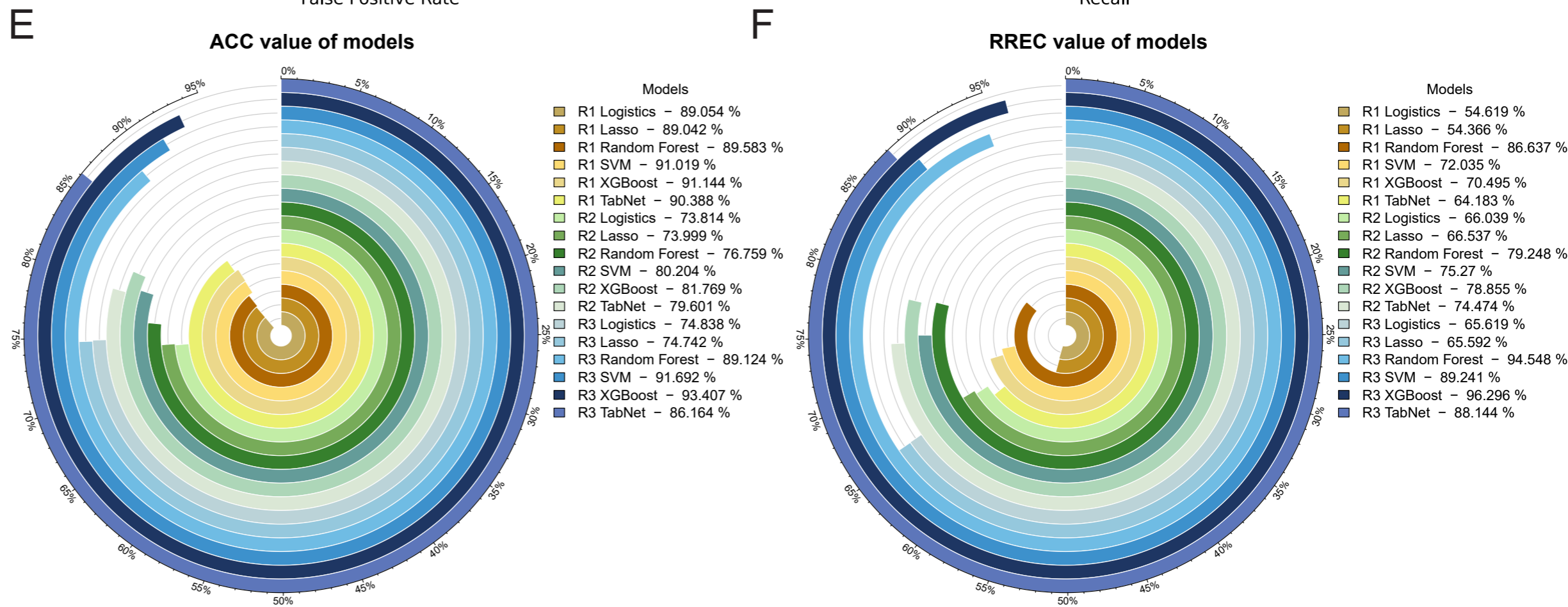

Supplement: Supplemental Information 2 — A-B represents the ROC curves of the models after weight adjustment and undersampling, indicating the AUC values. C-D represents the PR curves of the models after processing, indicating the AUPR values. E-F represent the accuracy and precision of each model after the three different methods, where R1 represents weight adjustment, R2 represents undersampling, and R3 represents oversampling. [file peerj-13-19078-s002.pdf]

A

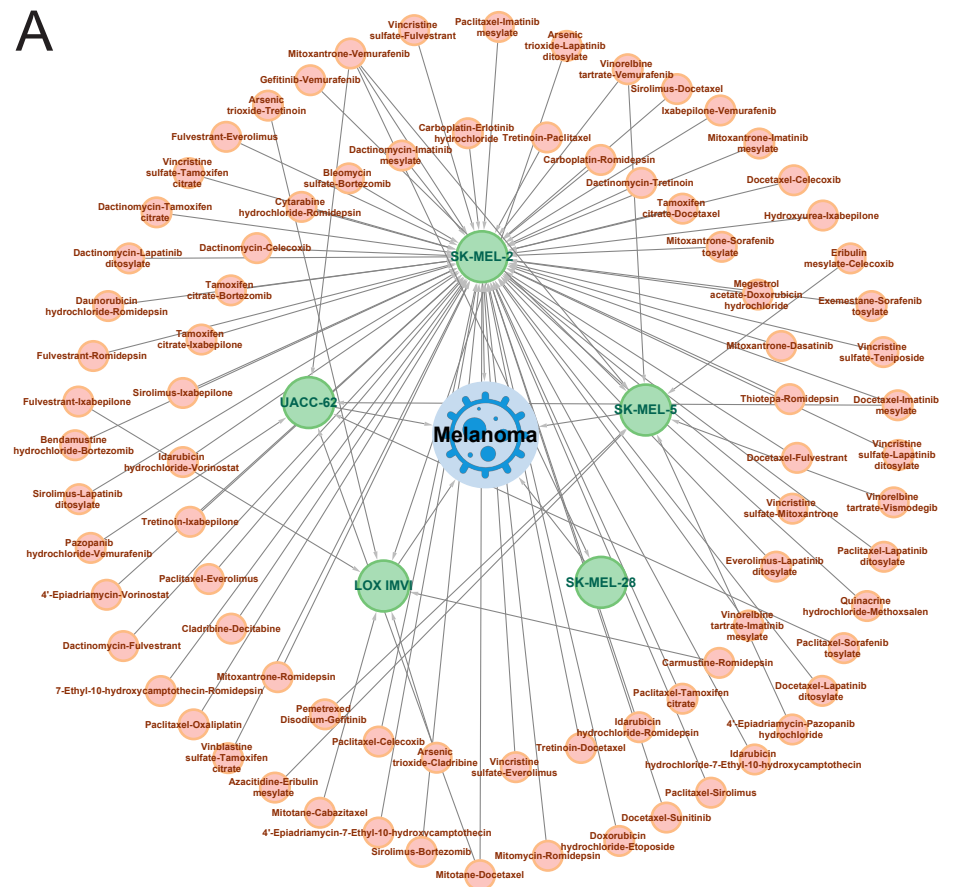

B

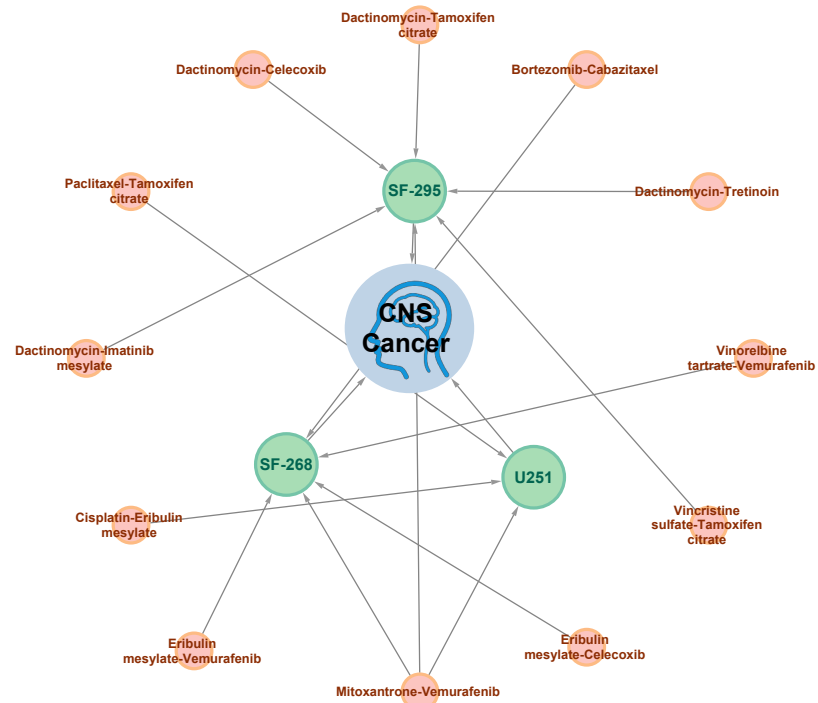

C

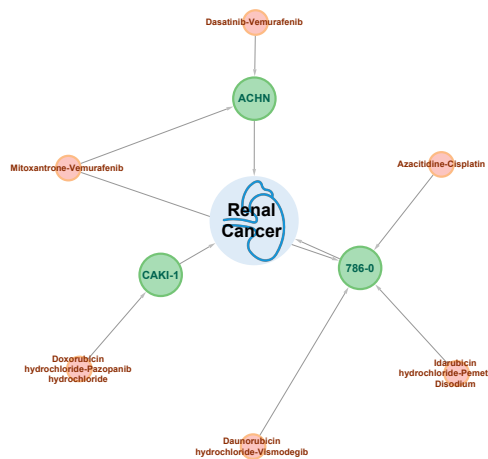

D

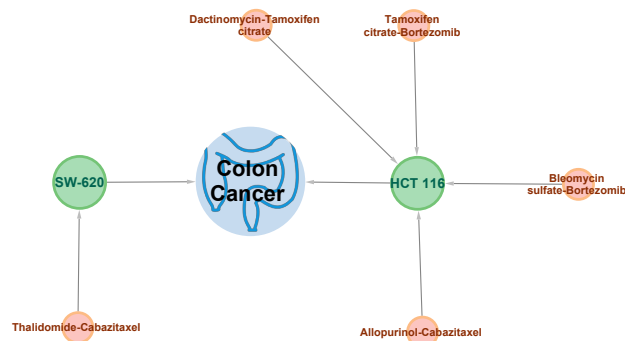

E

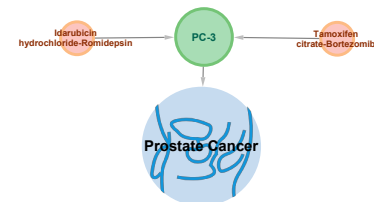

Supplement: Supplemental Information 3 — A-E represent the predicted drug combination networks that can act on melanoma, central nervous system cancer(CNS cancer), renal cancer, colon cancer, and prostate cancer-related cell lines. [file peerj-13-19078-s003.pdf]
